# Editor's Note on: Can a Y chromosome degenerate in an evolutionary instant? A commentary on Fong et al. 2023

**DOI:** 10.1093/gbe/evad152

**Published:** 2023-09-06

In the interest of transparency, we note for readers that we provided Deborah Charlesworth and co-authors an opportunity to clarify text in one section of their letter, including rewriting two sentences in the version of record of their accepted manuscript^1^. We made this decision after they read the reply^2^ from Lydia Fong and co-authors, which was simultaneously under review by the journal.

^1^ Deborah Charlesworth, et al. “Can a Y chromosome degenerate in an evolutionary instant? A commentary on Fong et al. 2023,” *Genome Biology and Evolution*, 2023; https://doi.org/10.1093/gbe/evad105

^2^ Julia Fong, et al. “Parsimony and Poeciliid Sex Chromosome Evolution,” *Genome Biology and Evolution*, 2023; https://doi.org/10.1093/gbe/evad128

